# International Brazilian of Urology is the Seventh Biggest Impact Factor (4.5) Among Urology and Andrology Journals in the World

**DOI:** 10.1590/S1677-5538.IBJU.2025.05.01

**Published:** 2025-07-25

**Authors:** Luciano A. Favorito

**Affiliations:** 1 Universidade do Estado do Rio de Janeiro Unidade de Pesquisa Urogenital Rio de Janeiro RJ Brasil Unidade de Pesquisa Urogenital - Universidade do Estado do Rio de Janeiro - Uerj, Rio de Janeiro, RJ, Brasil; 2 Hospital Federal da Lagoa Serviço de Urologia Rio de Janeiro RJ Brasil Serviço de Urologia, Hospital Federal da Lagoa, Rio de Janeiro, RJ, Brasil

**Figure 1:**
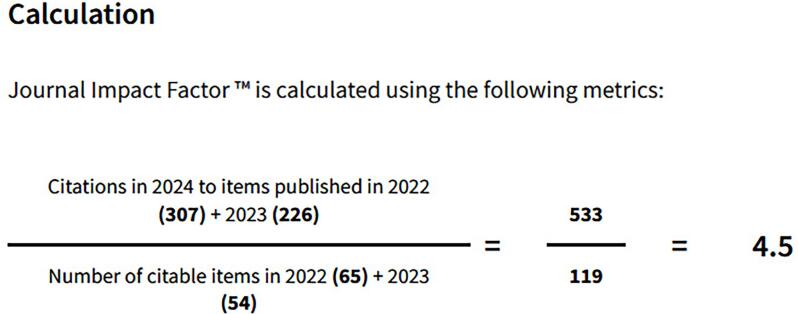
The figure shows the International Brazilian Journal of Urology calculation of the impact factor in 2024.

The 2025 September-October number of Int Braz J Urol, is very special. The International Brazilian Journal of Urology new impact factor just been released and we read a spectacular result: 4.5 (Figure-1). This significant increase in the impact factor in the last year is due to the rigorous and technical selection of published articles. Important papers in robotic surgery (1, 2), reconstructive surgery (3), sexual medicine (4), basic research (5), uro-oncology (6, 7), endourology (8) and neurourology (9) contributed greatly to increasing the impact factor due to the large number of citations they received.

**Figure 2:**
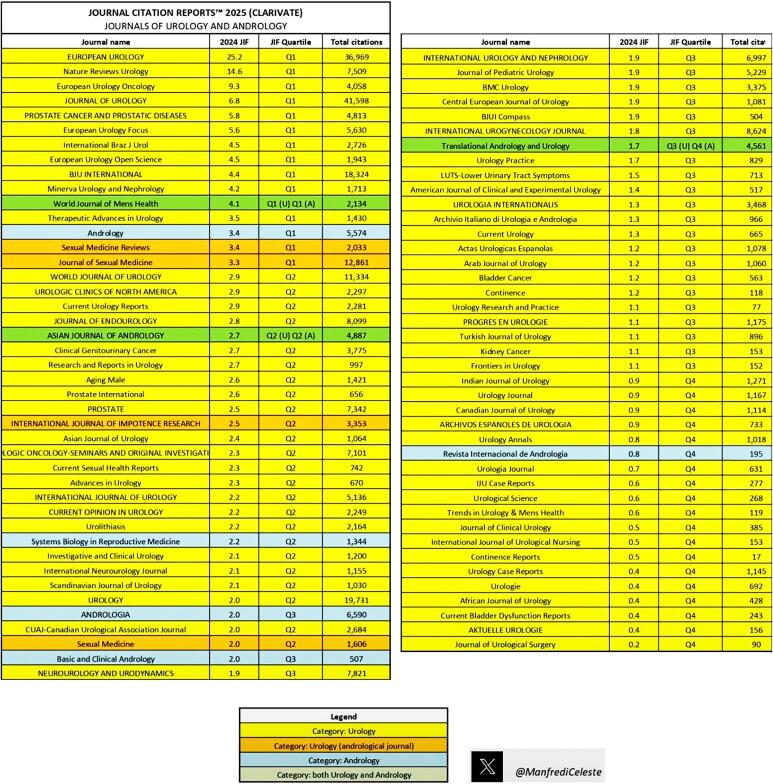
The figure shows the Jounal Citation Report 2025 (CLARIVATE) in Urology and Andrology. We can observe that the International Brazilian Journal of Urology with the new impact fator (4.5) is in seventh place among the 85 journals in this area, truly a great achievement

With this new impact factor, we are in seventh position among all andrology and urology journals in the world (Figure-2) and if we count the areas of nephrology and urology (which are related) we are the sixteenth largest impact factor among 133 journals analyzed. We are now on the same level as traditional urology journals. In less than six years of our tenure as editor-in-chief, we have achieved through hard and serious work quadripling the journal's impact factor and making the journal one of the most important in the field in the world.

What is striking is that the International Brazilian Journal of Urology is fully open access and does not charge a cent for authors to publish their articles. We are therefore the fully open-access journal with the greatest impact in the world in our area. We must celebrate this achievement and continue with our work. We are on the right track, and we will surely increase even more our impact factor and our relevance in the academic environment.

The Editor-in-chief expects everyone to enjoy reading the original contributions with a lot of interesting papers in this number.
